# Performance analysis of graphene modified asphalt and pavement performance of SMA mixture

**DOI:** 10.1371/journal.pone.0267225

**Published:** 2022-05-23

**Authors:** Peng Yong, Jianhua Tang, Fei Zhou, Rui Guo, Jie Yan, Tao Yang

**Affiliations:** 1 School of Civil Engineering and Architecture, Shaanxi University of Technology, Hanzhong Shaanxi, China; 2 Shool of Civil Engineering, Lanzhou University of Science &Technology, Lanzhou, Gansu, China; 3 School of Civil Engineering and Architecture, Hubei University of Arts and Science, Xiangyang, China; 4 Hubei Key Laboratory of Power System Design and Test for Electrical Vehicle, Hubei University of Arts and Science, Xiangyang, Hubei, China; Hacettepe Universitesi, TURKEY

## Abstract

The graphene modified asphalt used in this study was prepared based on a highway project in Gansu Province. In this paper, the high temperature rutting resistance, low temperature cracking resistance, and water stability of SMA-13 asphalt mixture with asphalt (AH-70), SBS modified asphalt and graphene rubber composite modified asphalt were tested and analyzed comparatively by the rutting test, Schellenberg binder drainage test, Cantabro test, freeze-thaw splitting test, and beam bending test. The results showed that the graphene modifier improved the asphalt’s ductility and softening point significantly, and 0.4g graphene content was the threshold and its corresponding mixture performance index. In the other tests under the same conditions, the high temperature and water stability of SMA-13 mixtures of graphene rubber modified asphalt were the best, followed by SBS modified asphalt mixture, and matrix asphalt mixture. Compared with wood fiber, graphene modifier had no significant effect on SMA-13 mixtures’ low temperature performance. The use of graphene modifiers can enhance the adhesion between asphalt and aggregate and its asphalt has good consistency and viscosity. When compared with matrix asphalt and SBS modified SMA-13 mixtures, the water and high temperature stability of graphene modified asphalt mixture is better.

## 1. Introduction

Asphalt mixtures are used widely in high grade highway pavement. Rutting deformation is the most prominent defects in early damage of asphalt pavement, which shortens the pavement’s service life [1–3]. Further, affected by the natural environment and worn by moving vehicles, asphalt pavement is prone to age with use. As a result, its water stability and ground temperature deformation capacity are weakened, which can lead to pavement damage, defects, and rutting disease that affect the quality of the pavement structure and its service life further [4–6]. SMA asphalt pavement has good durability and water stability, and performs well at high and low temperatures. Therefore, it is used widely in China. Unlike an ordinary dense asphalt mixture, SMA is a type of intermittent grading asphalt mixture formed with sufficient asphalt binder and asphalt mastic with a particular stiffness that fills the voids in coarse aggregate. The pavement structure’s stability overall depends on the adhesion of asphalt binder, so it is necessary to select high adhesion asphalt as the binder in an SMA asphalt mixture [7]. Because ordinary asphalt is a thermoplastic material that is highly sensitive to temperature, its mixture is prone to rutting damage at high temperatures and cracking at low temperatures, which thus affects the use of asphalt pavement. Therefore, it cannot meet the asphalt quality requirement of national economic development [8]. Asphalt modifier is more and more widely used in highway engineering, which is to improve the performance of asphalt binder and its mixture. Therefore, the research of new asphalt modifier has been paid more and more attention by engineers and technicians. Therefore, the engineers pay more and more attention to research new asphalt modifier [9]. Researchers found that the nano-materials with a layered structure in particular could effectively improve the anti-aging properties of asphalt mixtures [10, 11]. Graphene is a type of honeycomb-like, two-dimensional nanomaterial formed by the hybridization of C atoms via sp2 electronic orbits. Its unique structure and excellent performance make it a popular research topic in the field of materials, electronics, information, and so on [12]. The application of graphene in the field of civil engineering and research on the properties of graphene asphalt and other composites can not only help obtain better performance, but also provide a new direction for the functional application of graphene materials [13–16].

The SMA mixture is an asphalt mixture with a dense framework structure that has the characteristics of well wedging force between the coarse aggregate, the asphalt surface’s aggregate thickness, and low porosity [17–20]. It has good performance as well in rutting resistance, anti-sliding during rainfall, and resisting damage properties [21, 22], which researchers and technicians worldwide have appreciated and studied. Stone Matrix Asphalt is a hot asphalt mixture that was developed firstly in Germany during the mid-1960s and has been used in Europe for more than 20 years to provide better rutting resistance, and resist tire wear and slide [8]. SMA pavements have also been used in more than 28 states in the US [23, 24]. Because of its success in Europe and the US, the first SMA pavement was constructed in China in 1993. Since then, the use of SMA pavements has increased significantly and was promoted nationwide by the ministry of transport of China in 2002 [25, 26].

SMA is a gap-graded aggregate hot asphalt mixture that has a higher proportion of coarse aggregate, lower proportion of middle-size aggregate, and higher proportion of mineral filler than a dense-graded mixture and contains 70%-80% coarse aggregate, 6%-7% binder, and 8%-12% filler [27]. It provides an efficient network with a stable stone-on-stone skeleton, and the SMA has the characteristics of well wedging force among coarse aggregate and low porosity [28, 29]. There is a significant difference between SMA and continuous dense gradation asphalt mixtures, in that the SMA mixtures are stabilized stone with a dense skeleton and high content of asphalt, 6–7.5% of the total mixture’s weight. SMA pavement is quite capable of resisting rutting, and thus has been receiving increasing attention by engineers and technicians in the field of roads.

At present, the methods to improve the rutting resistance of asphalt pavement are mainly to modify the asphalt binder and asphalt mixture. The asphalt modifier widely used in highway engineering is SBS, which has been used for more than 20 years. However, based on the investigation, the modification method of SBS asphalt modifier is relatively single, which makes it difficult to improve the deformation resistance of asphalt pavement. Graphene is a widely used nano-material and attracted extensive attention in the road as asphalt modifier. In this paper, the road performance of AH-70^#^ matrix asphalt, SBS modified asphalt, and graphene rubber composite modified asphalt was analyzed and compared to determine the graphene modifier’s effect on the performance of asphalt and its SMA mixtures and provide reference for similar projects.

## 2. Experimental materials and analysis method

### 2.1. Properties of raw materials

#### ① Asphalt binder

AH-70^#^ petroleum asphalt produced by PetroChina Karamay Petrochemical Co., Ltd was selected as the base asphalt for the test. It is black and viscous in appearance, and its principal physical properties are shown in Table 1.

**Table 1:** The main technical performance of AH-70# asphalt.

Items		AH-70^#^ base asphalt
Penetration Value (25°C, 100g, 5s (/0.1mm		72
Softening Point /°C		49
Ductility (5cm∙min^-1^)/cm		100
Residue after	Mass Change /%	-0.018
rotating film	Remaining Penetration Value /%	69.4
heating	Remaining Ductility (10°C)/cm	12

#### ② Asphalt modifier

**Fig 1:**
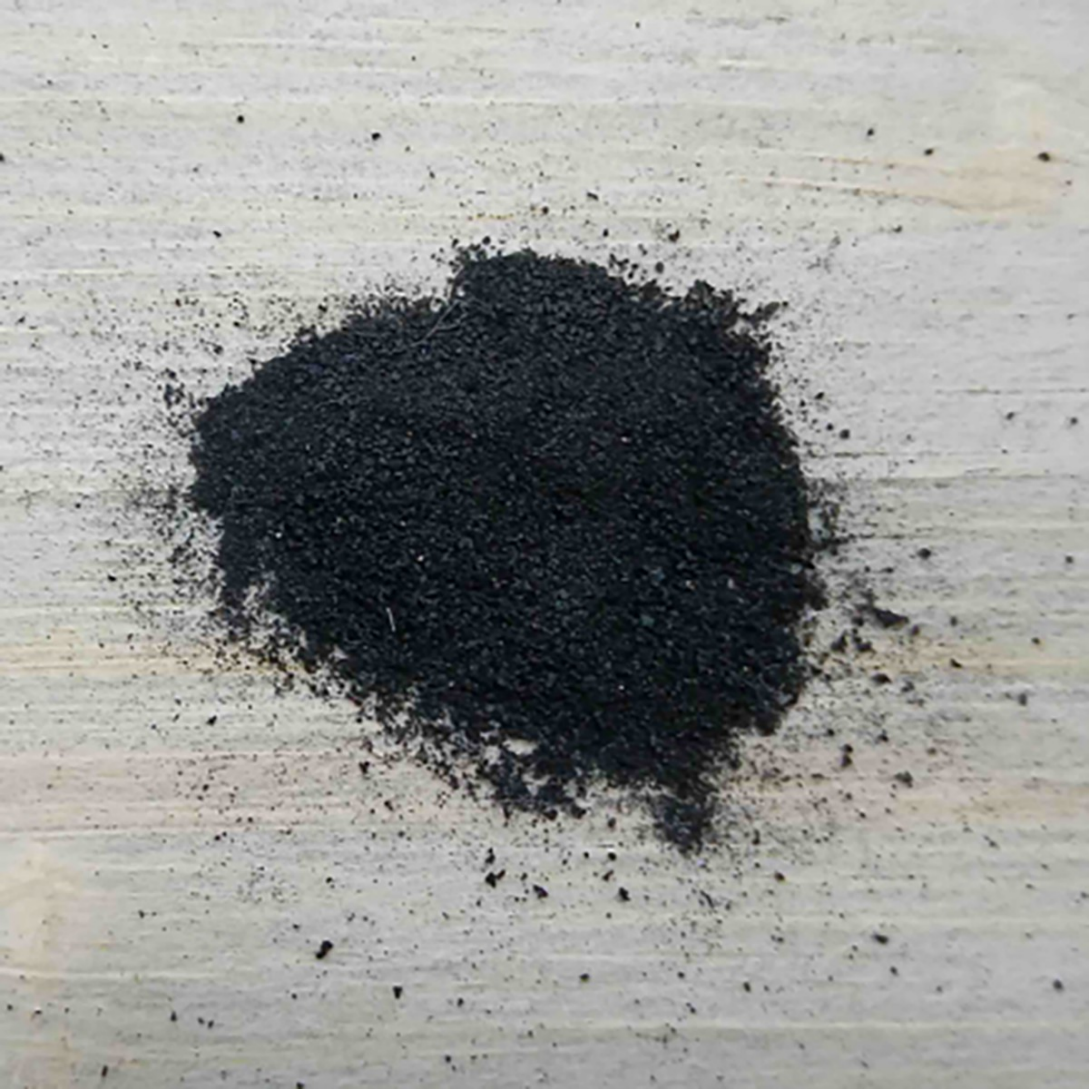
The surface morphology of graphene oxide.

**Fig 2:**
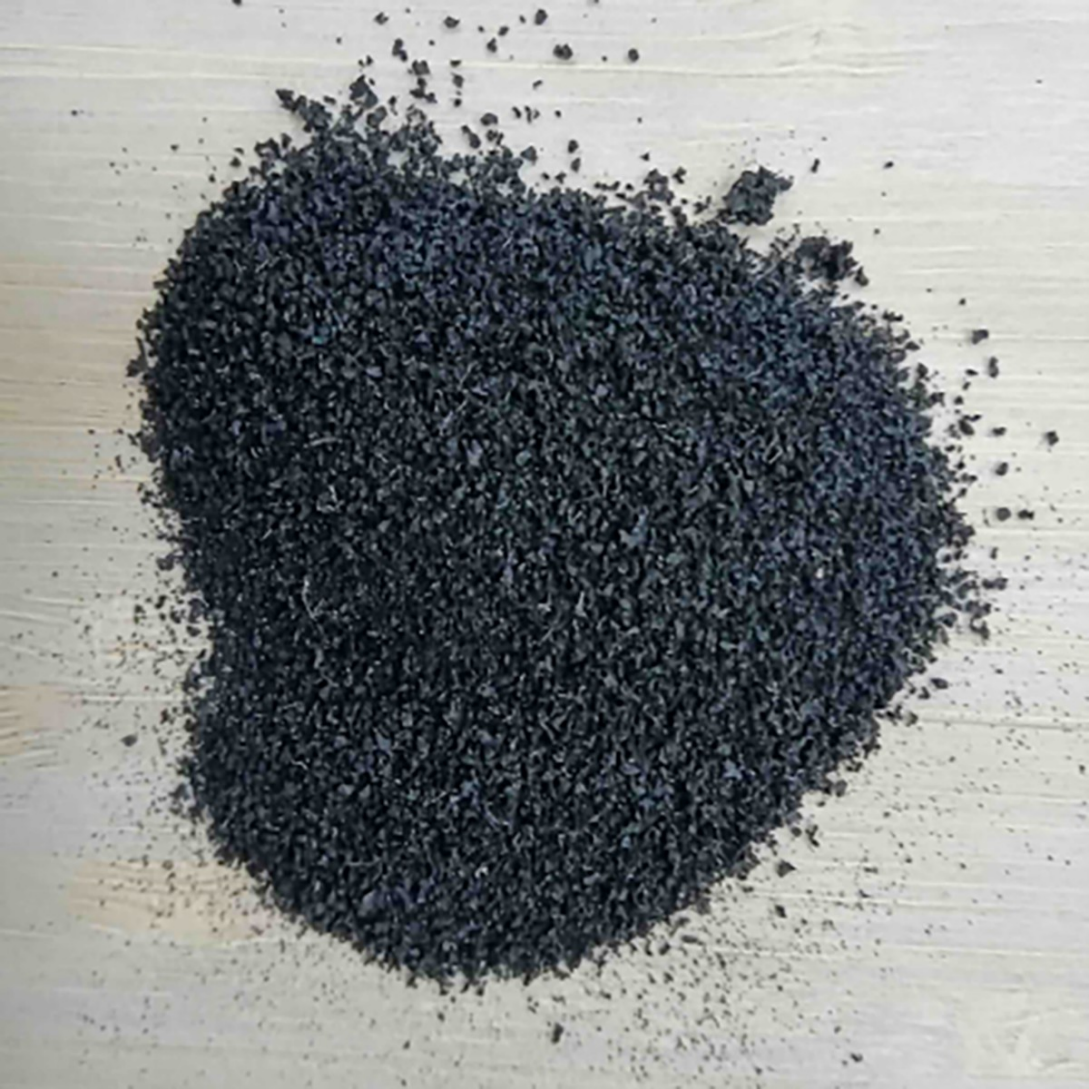
The surface morphology of rubber powder.

The graphene oxide used in the test is black powder (as shown in Fig 1). Its main physical properties are shown in Table 2 and the infrared spectrum test results are shown in Fig 2. The rubber used in the test is 40 mesh waste rubber powder produced by Sichuan Dujiangyan Huayi Rubber Co., Ltd. It is in the form of black particles, and its main physical properties are shown in Table 3, which has been based on the standard [30, 31]. In this study, the main component of the asphalt modifier for producing SBS modified asphalt is thermoplastic styrene butadiene rubber, and its appearance is linear and white stripe. The main indices are shown in Table 4.

**Table 2:** The main technical performance of graphene oxide.

Items	Measured Value	Items	Measured Value
Appearance	Gray-black powder	Specific Surface Area	182m^2^/g
Bulk Density	0.017g/ml	Thickness	One to three layers
Particle Size	10um	Hydrophilicity	Difficult to be wetted by water
Carbon Content	98%	Neck-Thickness Ratio	9500 on average
Water Content	≤2%		

**Table 3:** The main technical performance of rubber powder used.

Items	Measured Value	Technical Requirement
40 mesh Content /%	92	≥90
Combustion Residue /%	37	<38
Ash /%	4.5	≤10
Rubber Content /%	51	≥48
Fiber Content /%	0.5	<1
Water Content /%	0.6	<1

**Table 4:** The physical properties of asphalt modifier used.

Surface appearance	Density (g·cm^-3^)	Particle diameter (mm)	Melting point (°C)
White linear	0.78	3~6	142

#### ③ Coarse and fine aggregate

In view of the actual situation of the supporting project, the aggregate used in the test was taken from the project site. Its lithology is limestone gravel and manufactured sand. It was tested by the method specified in the Highway Engineering Aggregate Test Procedures [32] (JTG E42-2005). The main technical performance specifications are shown in Table 5 below and they met the quality requirements of aggregate as stipulated in the Technical Specification for Highway Asphalt Pavement Construction [15] (JTG F40-2004).

**Table 5:** The main technical performance of the aggregate used.

Items		Measured Value	Technical Requirement
Apparent Relative Density / (g·cm^-3^)	Gravel (10~15mm)	2.961	≥2.60
	Gravel (5~10mm)	2.982	
	Manufactured Sand (0~3mm)	2.718	≥2.50
Crush Value of Coarse Aggregate /%		16.0	≤22
Los Angeles Abrasion Loss of Coarse Aggregate /%		22	≤28
Los Angeles Polished Loss of Coarse Aggregate /%		42	≥36
Needle Flake Content of Coarse Aggregate/%	Gravel (10-15mm)	8.6	≤9
	Gravel (5-10mm)	9.4	≤11
Soft Particle Content /%		2.3	≤3
Sand Equivalent /%		76	≥60
Soundness/%		12	≥12

#### ④ Mineral powder

Limestone powder produced by Dunhuang Rongxing Building Materials Co., Ltd was selected for the test, and its primary technical performance indices are shown in Table 6 below. They all met the quality requirements of mineral powder for expressway asphalt mixture in the Technical Specification for Highway Asphalt Pavement Construction [33] (JTG F40-2004).

**Table 6:** The main technical performance of used mineral powder.

Items	Measured Value	Technical Requirement
Hydrophilic Coefficient	0.72	<1
Plasticity Index	3.3	<4
Appearance	No agglomerate	No agglomerate
Heating Stability	No color change	Actual test record
Water Content /%	0.4	≤1
Apparent Density/ (g/cm^3^)	2.674	≥2.50

#### ⑤ Mineral material

**Fig 3:**
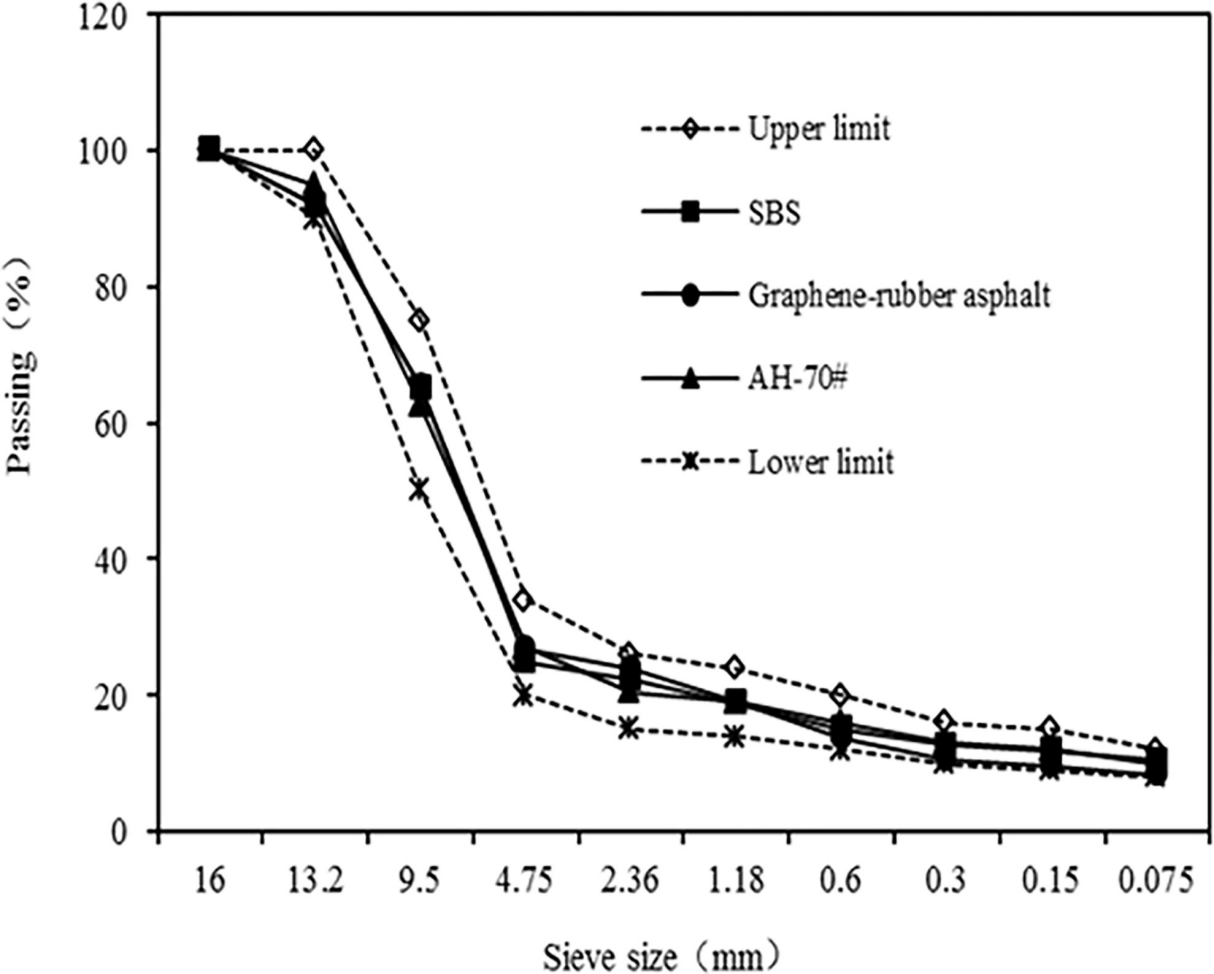
The gradation type of mineral aggregate of asphalt mixture.

Based on the actual condition of the supporting project, SMA-13 was selected as the asphalt mixture, and the proportion of the mixture was determined by the Marshall test. To analyze the graphene modifier’s effect on the road performance of different asphalt mixtures, three types of mixtures were chosen, AH-70^#^ matrix asphalt, SBS modified asphalt, and graphene rubber composite modified asphalt. The three kinds of mixtures’ gradations is nearly the same in the design of mineral aggregate gradation. The gradation composition is shown in Fig 3.

### 2.2. Test scheme

Firstly, it draws the research methods in literature Nos. 8 to 10 and the research findings of previous studies [34], and in view of the actual condition of the supporting project, Karamay AH-70^#^ petroleum asphalt was selected as the matrix asphalt, which was weighed accurately at an interval of 0.1g, and then was added to the matrix asphalt (weight 100g) to prepare modified asphalts with different graphene content. When the base asphalt was heated to 120C, graphene oxide powder was added to it, and then the container of the mixture was placed on a magnetic stirrer with a rotating speed of 3000r/min and a temperature of 100~120C to shear and stir for 6 hours, so the redox reaction was completed fully and the graphene oxide was dispersed evenly in the base asphalt to obtain graphene modified asphalt. Rubber powder was added to the modified asphalt and then stirred continuously for 45 minutes under the rotational speed and temperature above to make graphene rubber composite modified asphalt. Secondly, according to the requirements of Test Procedures for Asphalt and Asphalt Mixture in Highway Engineering [35] (JTG E20-2011), some major performance indices, such as the penetration value, softening point, and ductility, were tested on matrix asphalt AH-70^#^, SBS modified asphalt, rubber SBS modified asphalt, and graphene rubber modified asphalt to evaluate their aging properties. The graphene modifier’s effect on asphalt performance was also compared and analyzed. Finally, the high- and low-temperature performance, and water stability of the matrix asphalt mixture, SBS modified asphalt mixture, and graphene rubber modified asphalt mixture were tested to analyze the graphene modifier’s effect on the asphalt mixtures’ road performance.

## 3. Test results and analysis

### 3.1. Performance analysis of asphalt binder

#### 3.1.1. Effect of graphene content

**Fig 4:**
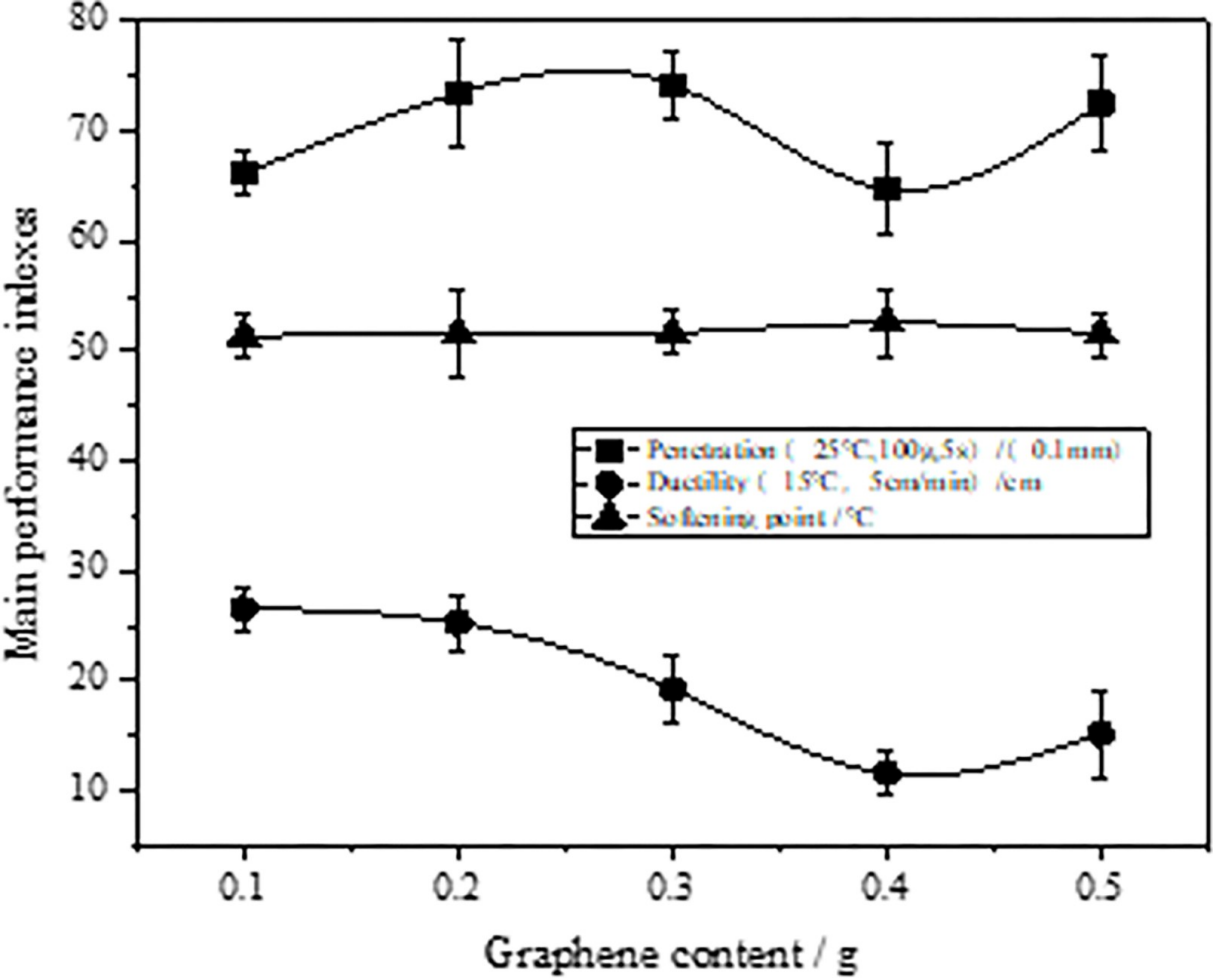
Relation between asphalt binder’s main performance indices and graphene content.

To analyze the graphene’s effect on the asphalt binders’ performance, modified asphalts with different graphene content were prepared according to the method described in test scheme 1.2. Then, their main performance indices were tested according to the operation requirements stated in Regulations [35] (JTG E20-2011). The results are shown in Fig 4.

It can be seen from Fig 4 that as the graphene in the matrix asphalt (AH-70^#^) increased, the asphalt’s ductility deceased first and then increased, and the relation curve between the two was similar to a parabolic curve. When the graphene modifier content was 0.4g, the corresponding modified asphalt’s ductility was the smallest, and the asphalt binder’s plasticity was the poorest. However, as the graphene content increased, the relation curve between the graphene content and the asphalt binder’s softening point showed an opposite trend. Further, when the graphene content was 0.4g, the asphalt binder’s softening point was highest, indicating that the modified asphalt had the best heat resistance. However, when the graphene content was 0.4g, the penetration value of its corresponding modified asphalt was the smallest, and the asphalt binder’s hardness and viscosity were greater. In summary, for AH-70^#^ matrix asphalt, when the graphene content was 0.4g, the asphalt binder’s adhesion and heat resistance were good, but its plasticity was poor. The high temperature stability, construction workability, and thermal storage stability of its corresponding asphalt mixture improved. Therefore, we concluded that 0.4g graphene content is a key value that affects the main performance indices of graphene modified asphalt and those of its corresponding asphalt mixture.

#### 3.1.2. Main properties of asphalt binde

To analyze different asphalt binders’ changes in penetration, ductility, and softening point, and the graphene modifier’s effect on the binders’ performance, we prepared graphene modified asphalt (with 0.4g graphene content) and graphene rubber composite modified asphalt (0.4g graphene content, 0.2% rubber content) according to the method described in test scheme 1.2 and the research findings in literature Nos. 7, 11, 12, and 13, as well as the previous results of our study. In accordance with the operating requirements of Regulations [35] (JTG E20-2011), the main indices of matrix asphalt (AH-70^#^), SBS modified asphalt, graphene modified asphalt, and graphene rubber composite modified asphalt were tested. The results are shown in Table 7.

**Table 7:** The index values of different types of asphalt binders.

Type of asphalt binder Technical requirement		70^#^matrix asphalt	graphene modified asphalt	rubber modified asphalt	graphene rubber composite modified asphalt	SBS modified asphalt
Penetration Value (25°C, 100g, 5s)/0.01mm		72	64.8	62	57	78
Ductility (5°C, 5cm·min^-1^)/cm		10	13.6	15	23	37
Softening Point /°C		49	52.6	65	83	83
135°C Brookfield Viscosity / (Pa·S)		0.310	0.58	2.42	2.7	2.143
Residue after rotating film heating	Mass Change/%	-0.018	-0.012	-0.091	-0.090	-0.06
	Penetration Ratio (25°C)/%	70.8	87	81	91	82.3
	Ductility 5°C	8	12	10	21	30

It can be seen from the test results in Table 7 that first, compared with 70^#^ matrix asphalt and rubber modified asphalt with no graphene added, graphene modified matrix asphalt and graphene rubber composite modified asphalt had lower penetration values, a decrease of 10% and 8.1%, respectively. However, their ductility values showed an opposite trend and increased 60% and 53.3%, respectively. The softening point is increased by 7.3% and 27.7%. Therefore, we concluded that the addition of graphene modifier in matrix asphalt and rubber modified asphalt can improve the ductility and softening point and reduce the penetration value significantly. The reason for this outcome is that the binder’s viscosity improved when graphene modifier was added to AH-70^#^ matrix asphalt and rubber modified asphalt, and stiffness and deformation resistance improved as well. However, the softening point did not meet the requirements.

Second, compared with AH-70^#^ matrix asphalt and rubber modified asphalt, the viscosity values of graphene modified matrix asphalt and graphene rubber composite modified asphalt increased by 87.1% and 11.6%, respectively. In addition, the increase in the former was more significant than in that of the latter. The explanation for this result is that the addition of graphene modifier to different types of asphalt binders improved the density of the asphalt’s inter-molecular hinges, leading to a denser inter-molecular structure and a more stable asphalt system that increased the asphalt binder’s viscosity thereby.

Third, the aging test suggested that the three types of asphalt binders’ weight all decreased. Compared with modified asphalt without graphene, graphene modified matrix asphalt and graphene rubber composite modified asphalt demonstrated smaller weight losses and larger penetration values. The ductility variation range (50C) of modified asphalts with graphene was smaller than that without before and after aging. Further, the variation range was smaller than that of SBS and rubber modified asphalt.

### 3.2. Road performance analysis of asphalt mixture

To analyze the graphene modifier’s effect on the performance of the asphalt mixture, including high and low temperature resistance, water stability, and water permeability, three types of asphalt mixtures were designed in the test, AH-70^#^ matrix asphalt mixture (SMA-13-AH), SBS modified asphalt mixture (SMA-13-SBS), and graphene rubber composite modified asphalt mixture (SMA-13-GO). SMA-13 was selected as the asphalt mixture for the test, and its mineral composition is shown in Fig 3.

#### 3.2.1. High temperature performance

**Fig 5:**
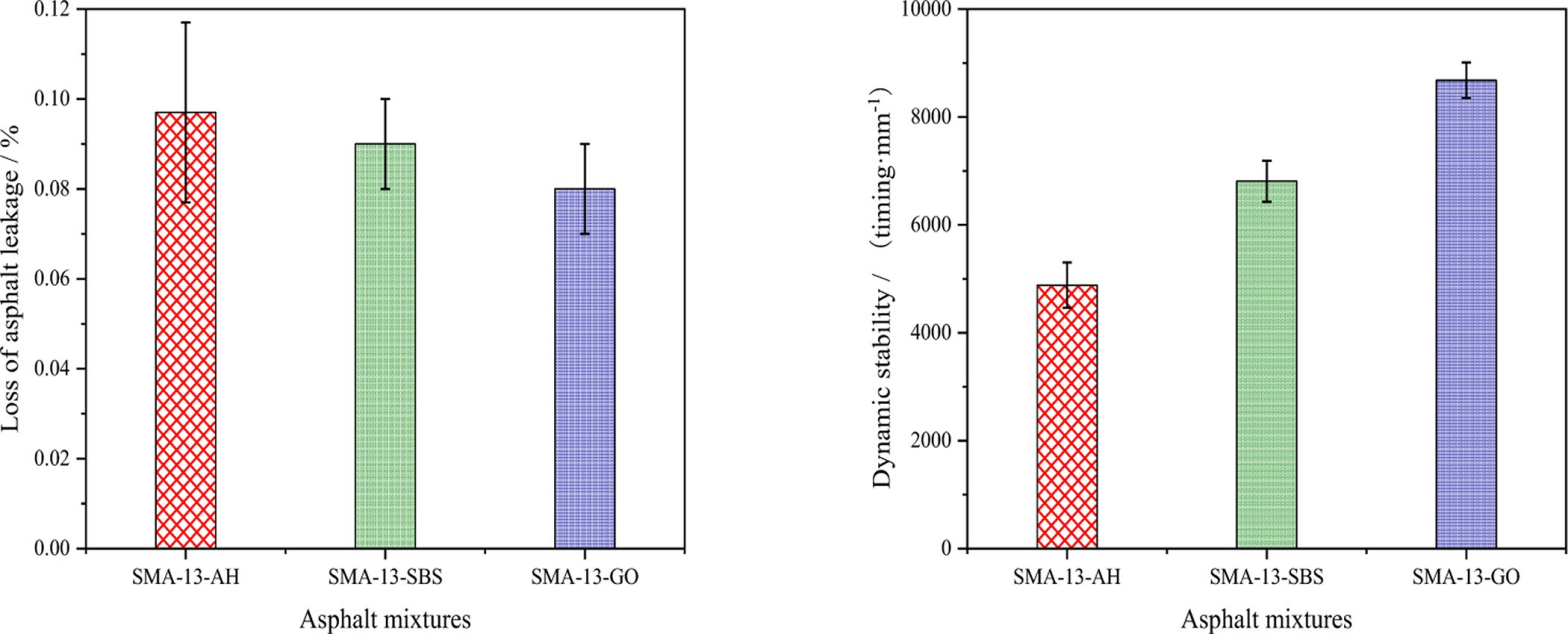
Asphalt mixtures’ high temperature performance. (a) Loss of asphalt leakage. (b) Dynamic stability.

We tested different asphalt mixtures’ asphalt precipitation and dynamic stability through the Schellenberg asphalt leakage test (test temperature: 1850C; holding time: 60min±1min) and rutting test (test temperature: 60±0.50C; time: 60min, wheel pressure: 0.7±0.05MPa) to analyze their performance at high temperature. It combined with the existing research findings [34], the asphalt aggregate ratio of the asphalt mixture used to make the specimens was 6.1%. Four parallel specimens were made for each type of asphalt mixture, and the mean value was taken as the test result. The results are shown in Fig 5 below.

It can be seen from Fig 5(A) that under the same test conditions, the asphalt leakage loss rates of the three types of mixtures was in the following order: RSMA-13-AH>RSMA-13-SBS>RSMA-13-GO, indicating that graphene modifier can improve asphalt mixtures’ high-temperature stability effectively. The reason for this is that the surface of graphene oxide contains more oxidation functional groups. After graphene modifier is added, it absorbs the asphalt mixture’s components and forms hydrogen bonds that produce van der Waals force, which can increase the molecular structure’s density, as well as the consistency and viscosity, and thus leads to a more stable system in asphalt binder. Adhesion between asphalt and aggregate is enhanced and the asphalt binder’s anti-stripping ability is improved accordingly. It has compared with SMA-13-AH and SMA-13-SBS, the asphalt leakage loss ratio of SMA-13-GO decreased by 17.5% and 11.1%, respectively.

It can be seen from Fig 5(B) that under the same test conditions, the three types of asphalt binder mixtures’ dynamic stability was in the order DSMA-13-GO>DSMA-13-SBS>DSMA-13-AH, indicating that SMA-13-GO exhibits better high temperature deformation resistance compared with the other two types of mixtures. This is because, on the one hand, adding graphene modifier to the asphalt mixture enhances the adhesion between the asphalt and aggregate particles, thereby improving the mixture’s deformation resistance overall; on the other hand, rubber particles in the asphalt mixture are dispersed in the voids between aggregate particles, which can isolate aggregate particles and reduce the pressure among them effectively, which prevents the particles from excessive extrusion and crushing that otherwise may destroy the mixture’s structure. In this way, the asphalt mixture’s flexibility and ability to recover from deformation are enhanced overall.

#### 3.2.2. Water stability

To analyze the graphene modifier’s effect on the asphalt mixture’s water stability, Marshall specimens of asphalt mixtures with different binders were made, with the asphalt-aggregate ratio of 6.1%. In strict accordance with the requirements of Regulations [35] (JTG E20-2011), we conducted several tests, including the Kentucky dispersion test, immersion Marshall test, and freeze-thaw splitting test to test the different asphalt mixtures’ immersion Marshall stability, freeze-thaw splitting strength, and dispersion rate to analyze their water stability and the graphene’s effect. The results are shown in Table 8.

**Table 8:** The water stability performance of 3 asphalt mixtures.

Type of Asphalt Mixture Items		SMA-13-GO	SMA-13-SBS	SMA-13-AH	The technical requirements
Immersion Dispersion Test	Loss Rate / %	5.50	5.50	6.19	≤15
Immersion Marshell Test	Stability / kN	9.18	9.05	8.22	-
	Flow Value / mm	4.00	4.80	5.20	-
	Residual Stability MS_0_ / %	92.40	91.05	87.51	≥85
Freeze-thaw Splitting test	Splitting Strength / MPa	0.78	0.67	0.61	-
	Splitting tensile strength ratio TSR / %	92.80	88.60	82.15	≥80

It can be seen from Table 8 that firstly, under the same gradation conditions, the immersion and dispersion loss rate of the three types of asphalt mixtures all meet the technical requirements of the Regulations [35] (JTG E20-2011). However, their anti-loose performances differ. SMA-13-SBS and SMA-13-GO are quite similar in anti-loose performance, while SMA-13-AH is poorer than the other two. The reason for this is that adding graphene modifier to SMA-13-GO improved the asphalt binder’s consistency and viscosity, enhanced the adhesion between asphalt and aggregate, and improved the mixture’s integrity as well. The incorporation of wood fiber additives in SMA-13-SBS has a reinforcement effect, which increased the mixture’s integrity and improved its anti-dispersion ability.

Secondly, when other conditions are the same, the three types of mixtures followed the same order in stability and residual stability in the immersion Marshall test, SMA-13-GO>SMA-13-SBS>SMA-13-AH. However, their flow values were in the opposite order. Compared with AH-70^#^ matrix asphalt and SBS modified asphalt, graphene rubber composite modified asphalt improved the asphalt mixtures’ water stability best for the same reason as in the analysis above. SMA-13-GO’s residual stability was 5.6%, 1.5% higher than that of the other two asphalt mixtures.

#### 3.2.3. Low temperature performance

**Fig 6:**
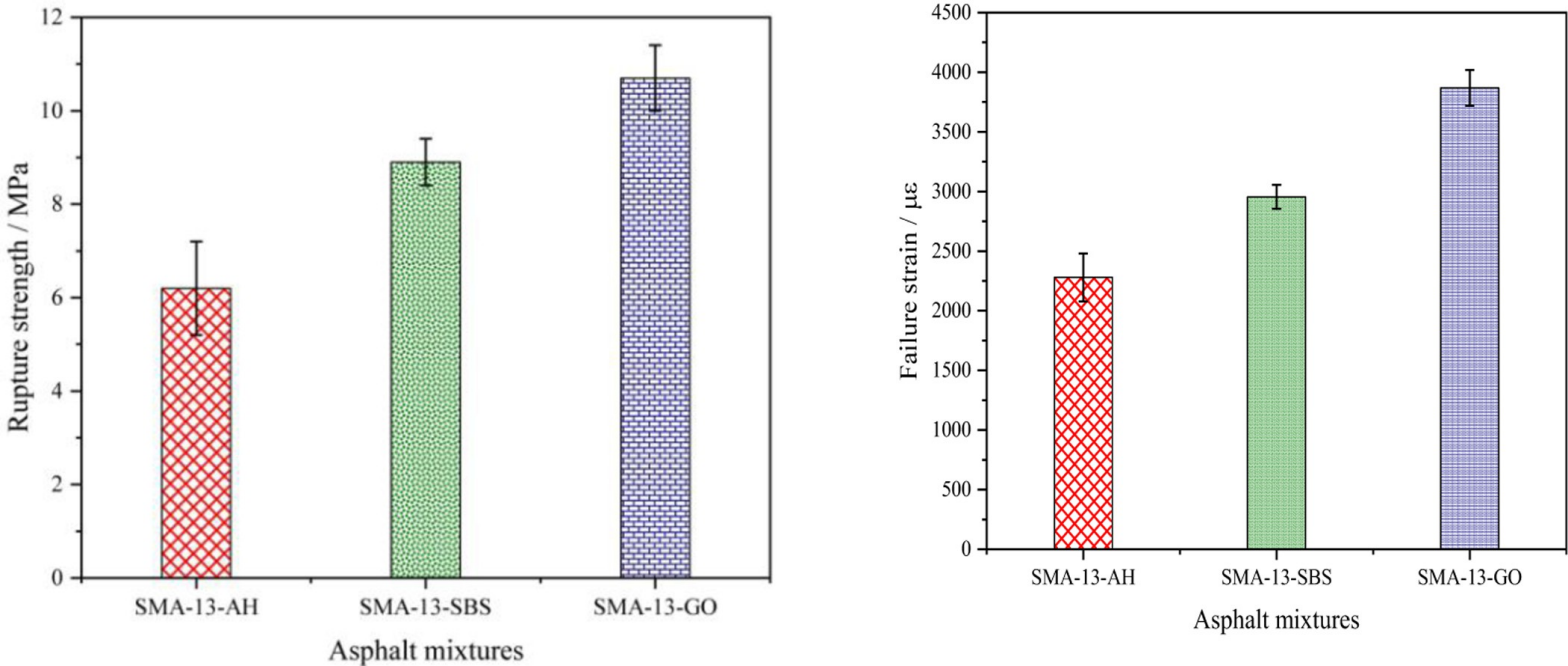
The asphalt mixtures’ low temperature performance. (a) Rupture strength. (b) Failure strain.

An asphalt mixture’s ultimate deformation capacity at low temperature reflects its low temperature performance. The bending strength and ultimate bending strain of SMA-13-AH, SMA-13-SBS, and SMA-13-GO were tested through the beam bending test, to conduct a comparative analysis of graphene modifier’s effect on asphalt mixtures’ low temperature performance. According to the operation requirements of the Regulations [35] (JTG E20-2011), four parallel specimens were made for each type of asphalt mixture and the mean value was taken as the test result. The results are shown in Fig 6.

As Fig 6 shows, when other conditions are the same, the failure strain of the three types of asphalt mixtures all met the requirements (≥2500με). Their failure strain and flexural tensile strength showed the same change trend, and followed the order SMA-13-GO>SMA-13-SBS>SMA-13-AH, which indicated that SMA-13-GO has good cracking resistance at low temperature. It can be seen from Fig 6(B) that the relative deviation in the failure strain between SMA-13-GO and SMA-13-SBS was 4.9%, indicating that adding graphene to an asphalt mixture can improve its low-temperature performance. However, compared with SBS modifier, graphene’s effect on the asphalt mixture’s low-temperature performance did not differ greatly, suggesting that graphene modifier has no significant effect on asphalt mixtures’ low-temperature performance.

### 3.3. Comparison of high and low temperature performance

In this section, the high and low performance of the asphalt mixtures (SMA-13-GO, SMA-13-SBS, SMA-13-AH) were compared based on the above research results. The change range of the asphalt mixture’s indices is based on the corresponding index value of SMA-13-AH asphalt mixture. The results are shown in Table 9.

**Table 9:** High and low temperature performance indices of 3 asphalt mixtures.

Asphalt mixture	Dynamic stability / timing·mm^-1^		Failure strain / με	
	value	change range	value	change range
SMA-13-AH	4882	-	2280	-
SMA-13-SBS	6809	39.47%	2955.5	29.63%
SMA-13-GO	8679	77.78%	3869.4	69.71%

It can be seen from Table 8 that Compared with SMA-13-AH asphalt mixture, the dynamic stability of SMA-13-SBS asphalt mixture is increased by 39.4%, and the failure strain is increased by 29.63%, which the increase of high-temperature performance index is larger than that of low-temperature performance index. For the SMA-13-GO asphalt mixture, it has compared with SMA-13-AH asphalt mixture that the dynamic stability and failure strain indexes of SMA-13-GO asphalt mixture increase by 77.78% and 69.711%, which shows that it can improve high and low temperature performance adding graphene modifier to the SMA-13 asphalt mixture, but compared with low temperature performance, the improvement of high temperature performance is more significant.

## 4. Fatigue life prediction of asphalt pavement

The fatigue failure of asphalt mixture is one of the key factors affecting the service life of asphalt pavement, and there are many researches on the fatigue performance of asphalt pavement at present [36]. In this paper, the fatigue life of the asphalt pavement corresponding selected asphalt mixture is calculated by using formulas (1)~(2) in the literature [37], which based on the actual situation of the project and the above testing results. The result is shown in Table 10.


$$N_{f1}=6.32\times 10^{15.96‐0.29\beta }k_{a}k_{b}k_{t1}^{‐1}\cdot {\left(\frac{1}{\varepsilon_{a}}\right)}^{3.97}{\left(\frac{1}{E_{a}}\right)}^{1.58}{\left(VFA\right)}^{2.72}$$ (1)

Where: *N*_*f1*_ is the fatigue cracking life of asphalt mixture layer (axle times). *Β* is the target reliability index. *k*_*a*_ is the adjustment coefficient of seasonal frozen soil area. *k*_*b*_ is the fatigue loading mode coefficient. *E*_*a*_ is the dynamic compression modulus of asphalt mixture at 20°C (MPa). *VFA* is the asphalt saturation of asphalt mixture (%). *k*_*t1*_ is the temperature adjustment coefficient. *ε*_*a*_ is the tensile strain at the bottom of the asphalt mixture layer (10^−6^).

**Table 10:** High and low temperature performance indices of 3 asphalt mixtures.

Asphalt mixture	*k*_*a*_	*t*_*T1*_	*E*_*a*_ */ MPa*	*VFA* / %	*N*_*f1*_ / axis·timming
SMA-13-AH	0.75	1.1	7800	75.9	0.82×10^7^
SMA-13-SBS	0.75	1.1	10087	77.4	1.13×10^7^
SMA-13-GO	0.75	1.1	12025	75.4	1.40×10^7^

It can be seen from Table 10 that under the same other conditions, the fatigue life of the asphalt mixture with asphalt modifier is significantly improved compared with that of without asphalt modifier. It has compared with SMA-13-AH asphalt mixture, the service life of SMA-13-SBS and SMA-13-GO mixture is increased by 37.80% and 70.31% respectively. The SMA-13-GO has the longest service life among the three asphalt mixtures, which shows that asphalt mixture added graphene modifier can improve the fatigue resistance and prolong the service life of asphalt pavement.

## 5. Conclusions

By testing the performance of different types of asphalt binders and asphalt mixtures, we analyzed graphene modifier’s effect on their properties and reached the following conclusions:

1. Compared with matrix asphalt (AH-70^#^), graphene modified asphalt and graphene rubber composite modified asphalt lost is less mass, and has a larger penetration ratio and smaller change in ductility (50C) after aging. A graphene content of 0.4g affects the main performance indices of asphalt and its asphalt mixture significantly.
2. Under the same test conditions, the three types of mixtures’ asphalt leakage loss rate is in the following order: RSMA-13-AH>RSMA-13-SBS>RSMA-13-GO, and the dynamic stability was in the order DSMA-13-GO>DSMA-13-SBS>DSMA-13-AH.
3. Under the same gradation conditions, SMA-13-GO had the greatest water stability, followed by SMA-13-SBS, and SMA-13-AH.
4. Adding graphene modifier to an asphalt mixture improved its low-temperature performance, but its effect did not differ significantly from that of other modifiers, such as wood fiber.

## References

[1] Wong W G, Han H, He G, “Rutting response of hot-mix asphalt to generalized dynamic shear moduli of asphalt binder,” Construction and Building Materials, vol. 18, pp. 399–408, 2004.

[2] Aravind K, Das A, “Pavement design with central plant hot-mix recycled asphalt mixes,” Construction and Building Materials, vol. 21, pp. 928–936, 2007.

[3] Xiao F, Amirkhanian S, Juang C, “Rutting resistance of rubberized asphalt concrete pavements containing reclaimed asphalt pavement mixture,” Journal of Materials in Civil Engineering, vol. 19, pp. 475–483, 2007.

[4] Javilla B, Mo L T, Hao F, “Multi-stress loading effect on rutting performance of asphalt mixtures based on wheel tracking testing,” Construction and Building Materials, vol. 148, pp. 1–9, 2017.

[5] Cong P, Xu P, Chen S, “Effects of carbon black on the anti-aging, rheological and conductive properties of SBS/ asphalt/ carbon black composites,” Construction and Building Materials, vol. 52, pp. 306–313, 2014.

[6] Serigos P A, Prozzi J A, Smit A F, “Evaluation of 3D automated systems for the measurement of pavement surface cracking,” Journal of Transportation Engineering, vol. 142, pp. 1943–540000841, 2016.

[7] Yu R E, Zhu X J, Zhang M R, “Properties and mechanism of graphene oxide/polyurethane composite modified asphalt mixture” Science Technology and Engineering, vol. 18, pp. 209–214, 2018.

[8] McNally T, “Polymer modified bitumen: Properties and characterization” Cambridge, Woodhead Publishing Limited, pp. 10–11, 2011.

[9] Jiang W T, Hao P W, Liu J W, “Effect of PVP modified graphene on rheological properties of SBS modified asphalt,” Journal of Building Materials, 2020-06-28, https://kns.cnki.net/kcms/detail/31.1764.TU.20200628.0849.004.html

[10] Liu K F, Zhu J C, Zhang X F, “Performance evaluation of graphene oxide modified asphalt and pavement performance of OGFC mixtures,” Journal of Chang’an University (Natural Science Edition), vol. 40, pp. 40–48, 2020.

[11] Cong P, Xu P, Chen S, “Effects of carbon black on the anti-aging, rheological and conductive properties of SBS/ asphalt/carbon black composites,” Construction and Building Materials, vol. 52, pp. 306–313, 2014.

[12] Weitz R T, Yacoby A, “Graphene rests easy,” Nature Nanotechnology, vol. 10, pp. 99–700, 2015. doi: 10.1038/nnano.2014.312 20924390

[13] Zhang X, Huang G, Zhou C, “Research status of graphene material in fields of asphalt composites,” Journal of Central South University (Science and Technology), vol. 56, pp. 1637–1644, 2019.

[14] Liu K, Zhang K, Junliang W U, “Evaluation of mechanical performance and modification mechanism of asphalt modified with graphene oxide and warm mix additives,” Journal of Cleaner Production, vol. 193, pp. 87–96, 2018.

[15] Zhu J C, Zhang K, Liu K F, “Performance of hot and warm mix asphalt mixtures enhanced by nano-sized graphene oxide,” Construction and Building Materials, vol. 217, pp. 273–282, 2019.

[16] Liu K, Zhu J, Wu C, “Experimental study on aging resistance of graphene oxide modified asphalt andits mixture,” Highway, vol. 2, pp. 225–230, 2020.

[17] Landsberg M, “Stone mastic asphalt trials in Ontario,” Transportation Research Record, 1993.

[18] Zheng-qi Z, Run-pu Q, Deng-liang Z, “Study of stone mastic asphalt performance,” China Journal of Highway and Transport, vol. 14, pp. 13–14, 2001.

[19] Ji-cheng H, Xiang Z, Peng H, “Systemic design and construction technology of stone matrix asphalt mixture,” Journal of Tong Ji University (Natural Science), vol. 34, pp. 69–73, 2006.

[20] Sharma V, Goyal S, “Comparative study of performance of natural fibres and crumb rubber modified stone matrix asphalt mixtures,” Canadian Journal of Civil Engineering, vol. 33, pp. 134–139, 2011.

[21] Dong-yu N, Sen H, Ou-ming X, “Research on the performance of SMA mixture modified by recycled LDPE and SBS,” Journal of Wuhan University of Technology, vol. 36, pp. 49–53, 2014.

[22] Loizos A, Papavasileiou V, and Prapidis A, “Laboratory evaluation of SMA mixtures,” Cell, vol. 60, pp. 565–575, 2002.

[23] Ahmadinia E, Zargar M, Karim M R, Abdelaziz M, Shafigh P, “Using waste plastic bottles as additive for stone mastic asphalt,” Materials & Design, vol. 32, pp. 4844–4849, 2011.

[24] Al-Hadidy A I, Tan Y Q, “Mechanistic analysis of ST and SBS-modified flexible pavements,” Construction & Building Materials, vol. 23, pp. 2941–2950, 2009.

[25] Behnood A, Ameri M, “Experimental investigation of stone matrix asphalt mixtures containing steel slag,” Scientia Iranica, vol. 19, pp. 1214–1219, 2012.

[26] Meng S T, Wei D X, “Research on anti-rutting performance of SMA asphalt pavement,” Journal of Highway and Transportation Research and Development, vol. 22, pp. 5–8, 2005.

[27] Austroads, “Technical note 16. Stone mastic asphalt, ARRB,” Transport Research, 2004.

[28] Asi I M, “Laboratory comparison study for the use of stone matrix asphalt in hot weather climates,” Construction & Building Materials, vol. 10, pp. 982–989, 2006.

[29] Zhou W, Huang X, Wang L, “Study on the void reduction behavior of porous asphalt pavement based on discrete element method,” International Journal of Pavement Engineering, vol. 18, pp. 285–291, 2017.

[30] General Administration of quality supervision, inspection and Quarantine of the people’s Republic of China. GB/T 14853.2–2002, “Rubber compounding ingredients-Carbon black pelletized-Determination of fines content,” Beijing: China Standards Press, pp. 1–2, 2002.

[31] IX-ISO. ISO 1138–2007, “Rubber compounding ingredients-Carbon black-Determination of sulfur content,” 2007.

[32] Institute of Highway Science, Ministry of Communications. JTG E42-2005, “Test methods of aggregate for highway engineering,” Beijing: China Communication Press, pp. 7–104, 2005.

[33] Institute of Highway Science, Ministry of Communications. JTG E42-2005, “Technical specifications for construction of highway asphalt pavements,” Beijing: China Communication Press, pp. 17–21, 2004.

[34] Guo R, “Analysis of influencing factors on rutting resistance of stone matrix asphalt mixtures,” Scientia Irania, vol. 27, pp. 1039–1049, 2020.

[35] Ministry of Transport of the People’s Republic of China. JTG E20-2011, “Standard test methods of bitumen and bituminous mixtures for highway engineering,” Beijing: China Communication Press, pp. 10–30, 2011.

[36] Liu F, Xu X Q, Zhang G M, “Influence of asphalt mixture composition difference on fatigue life of asphalt pavement structure,” Journal of Wuhan University of Technology, vol. 45, pp. 930–934, 2021.

[37] Ministry of Communications of the People’s Republic of China, Specifications for design of highway asphalt pavements JTG, Communication Press, Beijing, China, 2017, pp. D50–2017.

